# The Impact of Aerobic Exercise on Mood Symptoms in Trauma-Exposed Young Adults: A Pilot Study

**DOI:** 10.3389/fnbeh.2022.829571

**Published:** 2022-05-25

**Authors:** Allison L. Mizzi, Margaret C. McKinnon, Suzanna Becker

**Affiliations:** ^1^Department of Psychology, Neuroscience and Behaviour, McMaster University, Hamilton, ON, Canada; ^2^Department of Psychiatry and Behavioral Neurosciences, McMaster University, Hamilton, ON, Canada; ^3^Mood Disorders Program, St. Joseph’s Healthcare, Hamilton, ON, Canada; ^4^Homewood Research Institute, Guelph, ON, Canada

**Keywords:** trauma, aerobic exercise, physical activity, PTSD, mood, emotion regulation

## Abstract

**Introduction:**

Physical activity has beneficial effects on mood in both healthy and clinical populations. Emerging literature suggests that physical activity may benefit psychological symptoms, such as depressive mood, in those with post-traumatic stress disorder (PTSD). It is estimated that 76% of Canadians have experienced a traumatic event during their lifetime (Van Ameringen et al., 2008). Thus, there is a large proportion of the population that does not meet criteria for PTSD but may still suffer from trauma-related symptoms such as depression and require support for their mental health. The current pilot study aimed to evaluate the impact of an aerobic exercise intervention on mood symptoms in trauma-exposed young adults.

**Methods:**

Twenty-five low active young adults with subclinical trauma symptoms but no current or past diagnosis of PTSD were recruited. Participants were randomly assigned to participate in an 8-week exercise intervention group or a waitlist control group. Mood symptoms were assessed before and after the intervention. In addition, measures of aerobic fitness, trauma symptoms, emotion regulation, and trait mindfulness were assessed at both time points.

**Results:**

The exercise intervention was effective at inducing the expected improvements in aerobic fitness. Overall, the exercise group had a significantly greater decrease in mood symptoms across the intervention compared to the waitlist control group.

**Conclusion:**

The current pilot study is the first to evaluate the impact of aerobic exercise on mood in trauma-exposed young adults. An 8-week intervention significantly reduced mood symptoms in exercisers relative to waitlist controls. Our results are consistent with previous research indicating that physical activity reduced depressive symptoms in those with PTSD (Rosenbaum et al., 2015b). Importantly, we extend these findings to individuals with subclinical or undiagnosed PTSD symptoms, where exercise may be an effective intervention to improve mood and manage or prevent further decline in mental health in those at risk of developing PTSD.

## Introduction

Post-traumatic stress disorder (PTSD) may develop following exposure to a traumatic event. Approximately nine percent of Canadians will develop this condition within their lifetime (Van Ameringen et al., 2008). PTSD consists of four main symptom clusters: re-experiencing symptoms such as upsetting memories and flashbacks, avoidance symptoms such as avoidance of thoughts, people, or places related to the traumatic event, alterations in cognition and mood such as negative beliefs about oneself and the world or persistent negative mood, and alterations in arousal or reactivity such as irritability, hyperarousal, or difficulty concentrating (American Psychiatric Association, 2013). Importantly, these symptoms are typically very distressing and may be associated with functional impairment (American Psychiatric Association, 2013). In addition to these psychological manifestations of illness, individuals with PTSD exhibit higher rates of often dangerous physical health conditions such as obesity, diabetes, cardiovascular disease, and metabolic conditions (Rosenbaum et al., 2015b).

While about nine percent experience PTSD in their lifetime, approximately 76 percent of Canadians experience a traumatic event during their lifetime. Exposure to a traumatic event serves as a risk factor for negative psychological consequences. Trauma-exposed college students are at elevated risk for and develop more symptoms of mental health concerns, such as depression, self-harm, substance abuse, and anxiety (Vrana and Lauterbach, 1994; Flood et al., 2009; Barry et al., 2014). Traumatic event exposure is associated with greater medical symptoms and a greater number of physician’s office visits as compared to those who have no history of trauma (Boyraz et al., 2016; Artime et al., 2019). Critically, trauma exposure is associated with similar impairments in social and occupation functioning to PTSD (Zlotnick et al., 2002; Sienkiewicz et al., 2020) as well as rates of comorbid mental health disorders and incidence of suicidal ideation (Marshall et al., 2001). Thus, there is a sizable proportion of the population that has been exposed to trauma, does not meet criteria for a diagnosis of PTSD, but that may experience subthreshold PTSD symptoms and suffer a myriad of negative mental and physical health consequences because of this exposure. This is an important population for study given that treatment for a trauma-related disorder such as PTSD requires specific criteria to be met under guidance of the Diagnostic and Statistical Manual of Mental Disorders (DSM-5). Individuals not meeting the specific profile listed in the DSM-5 but suffering from subclinical symptoms related to trauma exposure have limited access to evidence-based resources to manage their mental health. Thus, accessible and alterative evidence-based resources are required to meet the needs of this unique population.

A common psychological symptom associated with both PTSD and trauma exposure is depressed mood. A history of traumatic event exposure is a substantial risk factor for depression (Youssef et al., 2013). Depression is one of the most common comorbid conditions within PTSD, with approximately 30–50% of individuals with PTSD also meeting diagnostic criteria for Major Depressive Disorder (MDD) (Angelakis and Nixon, 2015). Similarly, those exposed to a traumatic event are more likely to have experienced depressive symptoms in the past year, and to have been diagnosed with a depressive disorder (Artime et al., 2019). Prior research has found that lifetime prevalence of a major depressive episode in those with trauma exposure was greater than 35% (Alim et al., 2006). Critically, trauma-exposed individuals experiencing depression are at risk for worsening mental health and lower levels of functioning, given the profound negative impact of mood symptoms on physical, social, occupational, and emotional health (Fried and Nesse, 2014).

Traditional psychological treatments for trauma-related disorders include Prolonged Exposure Therapy (PE) and Cognitive Processing Therapy (CPT). Both PE and CPT apply a cognitive-behavioral perspective to address traumatic memories, thoughts, feelings, and behaviors associated with the traumatic experience. In PE, patients are repeatedly guided through recall of traumatic memories for deep processing and reducing negative thinking patterns and beliefs that once maintained trauma-related symptoms (Lancaster et al., 2016). CPT focuses on the cognitive component of PTSD by targeting maladaptive thinking patterns that contribute to PTSD and facilitating processing areas in their life affected by the trauma, such as trust, control, and safety (Lancaster et al., 2016). In addition to psychological therapy, pharmacological treatment is often a component of PTSD treatment. Although evidence-based psychological and pharmacological treatments are effective in treating symptoms of PTSD (Lancaster et al., 2016), there are significant barriers associated with these options. Psychological treatment for PTSD can be very challenging to receive for individuals who lack the financial resources, and/or easy access to a clinic or institution with trained trauma therapists to offer this specialized treatment. In addition to these barriers, waitlists for free, publicly funded psychological treatment in Ontario, Canada are often a year or more in length, which is disheartening for those struggling from distressing symptoms and functional impairment (Gratzer and Goldbloom, 2016). Importantly, access to evidence-based, publicly funded treatment is based on psychodiagnostics assessment and diagnosis with PTSD. However, this process excludes individuals with trauma exposure and subclinical symptoms, thus barring such individuals from treatment to manage the symptoms, which can be similarly distressing and impairing to those with a diagnosis of PTSD.

This need for more accessible treatments and resources within mental health care has prompted greater research into alternative evidence-based options for those unable to access care. Exercise may pose an effective adjunctive or, in early stages of illness, alternate, treatment option for some mental health conditions, including PTSD and MDD. Aerobic exercise has demonstrated effectiveness in treating symptoms in patients recurrent Major Depressive Disorder (Mota-Pereira et al., 2011) at a level comparable to antidepressant medication (Blumenthal et al., 2007). Exercise also appears to have benefit for anxiety symptoms and disorders (Herring et al., 2014). Specifically, a meta-analysis of 104 studies examining the effects of exercise on anxiety among healthy populations indicated that exercise training reduced both state and general anxiety significantly (Petruzzello et al., 1991). Similarly, in patients diagnosed with Generalized Anxiety Disorder (GAD), both high and low intensity aerobic exercise had a moderate to large effect on worry and anxiety symptoms (Herring et al., 2011; Plag et al., 2020). In addition, significant reductions in anxiety scores have been observed in patients diagnosed with panic disorder with agoraphobia who exercised three times per week for 8 weeks (Martinsen et al., 1989). This effect of exercise on anxiety extends to non-aerobic forms of exercise, such as yoga and resistance exercise, as well (Herring et al., 2014). Additionally, regular physical activity, such as walking, cycling, or swimming, produces a host of physical health benefits including reduced risk of cardiovascular disease, diabetes, and obesity among ostensibly healthy individuals (Penedo and Dahn, 2005; Warburton et al., 2006).

Given the strong impact of exercise on mood and anxiety for individuals experiencing mental health concerns, it is unsurprising that research examining exercise for traumatized populations is growing. A randomized control trial examined the effects of a 2-week aerobic exercise program on symptoms for those with a diagnosis of PTSD. Aerobic exercise significantly reduced patients’ PTSD symptoms from pre-intervention levels (Fetzner and Asmundson, 2015). Another study examined the impact of usual care (psychotherapy) alone vs. in combination with a 12-week exercise program on mood and PTSD symptoms among individuals with PTSD (Rosenbaum et al., 2015a). Here, as compared to the usual care only group, the combined group demonstrated significantly greater reductions in both depressed mood and PTSD symptoms. Moreover, the combined group exhibited significant reductions in body fat percentage, waist circumference, and sitting time per weekday—all risk factors for poor physical health and chronic diseases such as cardiovascular disease, obesity, and diabetes (Rosenbaum et al., 2015b). A recent review of the literature on physical activity and PTSD concluded that physical activity has demonstrated effectiveness in reducing mood symptoms in those who are diagnosed with PTSD as well as improving symptoms of mental health conditions that often accompany trauma, such as MDD (Oppizzi and Umberge, 2018). One meta-analysis (Rosenbaum et al., 2015b) examined four randomized control trials investigating the effect of physical activity interventions on PTSD and depressed mood. It emerged that physical activity was effective at reducing mood symptoms in people with PTSD.

The evidence reviewed above indicates that physical activity may be effective at improving mood in those diagnosed with PTSD, MDD, and anxiety disorders. However, no research to date has examined the impact of physical activity on mood in trauma-exposed populations without a clinical diagnosis, who have subclinical levels of symptoms and are at elevated risk of developing PTSD and/or MDD. Exercise may be a particularly appropriate and accessible option for this population, as it is accessible and cost effective and has an established effectiveness in improving mood. As noted earlier, 76% of Canadians report being exposed to traumatic events and 9% develop PTSD (*Post-traumatic Stress Disorder*, 2013), Therefore, there is likely to be a large proportion of individuals experiencing mood-related symptoms from their exposure, but who either do not meet diagnostic criteria for PTSD or simply have not been diagnosed due to lack of access to mental health care. Critically, this trauma-exposed population merits further investigation, as these individuals experience distressing and functionally impairing symptoms associated with both mood and physical health. Given that exercise training is a cost-effective, widely available, and non-resource intensive activity with growing evidence that it improves the mental health of clinically diagnosed individuals with PTSD, exercise stands as a potential means of managing and/or reducing mood-related symptoms in those who are trauma exposed, and could be a critical intervention for preventing the transition to PTSD or MDD.

The current pilot study examined the impact of an aerobic exercise intervention on mood-related symptoms in trauma-exposed young adults with subclinical levels of trauma symptoms. Accordingly, our main hypothesis was that the exercise intervention would reduce mood-related symptoms in trauma-exposed young adults. A second outcome measure was the change in aerobic fitness associated with exercise. This was a critical manipulation check to verify that the exercise intervention was effective.

Secondary outcome variables included measures of trauma symptoms, emotion regulation, and trait mindfulness. Prior research indicates that trait mindfulness, the tendency to pay attention to the present moment in an open and non-judgmental manner (Brown and Ryan, 2003) and emotion regulation, the ability to use adaptive strategies to manage emotions (Gross, 1998), are factors which impact mental health and wellbeing (Shapiro et al., 2011; Hu et al., 2014). Moreover, both trait mindfulness (Mothes et al., 2014) and emotion regulation skills (Bernstein and McNally, 2018) are positively influenced by exercise. Thus, we included these secondary measures to explore the potential impact of exercise on changes in fitness, trauma symptoms, trait mindfulness, and emotional regulation. It was hypothesized that those in the exercise group would display greater improvements in fitness, reduced trauma symptoms, as well as increases in emotion regulation and trait mindfulness relative to controls.

## Materials and Methods

### Participants

Twenty-five adults between the ages of 18–30 years from McMaster University were recruited, provided informed consent, and compensated for their time *via* course credit in their introductory psychology course and/or monetary incentive. Participants with a self-reported current diagnosis of PTSD, complex trauma, or dissociative disorder were excluded from the study based on a screening questionnaire. Interested participants were administered the Get Active Questionnaire (Canadian Society for Exercise Physiology, 2017) to verify that they had no contraindications to participating in exercise safely and were currently participating in less than 150 min of moderate-to-vigorous physical activity per week. Potential participants were also administered the PTSD Checklist for DSM-5 (PCL-5) (Blevins et al., 2015) to assess trauma-related symptoms. Those with scores between 4 and 25 on the PCL-5 were included based on clinical recommendations by a clinician-scientist at McMaster University (MCM) who is an expert in trauma research and clinical treatment. Potential participants were also asked to verify that they had access to the following equipment to be used within the study: a standard, indoor (20.3 cm height per stair) set of stairs, a level, hazard-free indoor space of approximately 3 m by 3 m to be used safely for exercise, closed-toed running or exercise shoes, a digital bathroom scale, and a device with a webcam, microphone, and internet connectivity. Based on previous research and mental health and exercise-based intervention studies run in the lab, an approximate sample size of 12 per group was required. The study was approved by the McMaster Research Ethics Board, #4902.

### Materials

#### Questionnaires

##### Demographic Information Questionnaire

This questionnaire collects basic demographic information, specifically, age and gender.

*PTSD Checklist (PCL-5).* The PCL-5 is a 20-item questionnaire assessing severity of trauma-related symptoms (Blevins et al., 2015). The questionnaire poses questions about how much the participant was bothered by a specific symptom or experience in the past month, for example, “In the past month, how much were you been bothered by repeated, disturbing, and unwanted memories of the stressful experience?” Each item is rated on a 5-point Likert scale ranging from “Not at all” to “Extremely.” A total symptom severity score can be calculated by adding scores for each item across the questionnaire (range = 0–80). The PCL-5 has been demonstrated to have satisfactory internal consistency and convergent validity (Sveen et al., 2016).

##### Depression Anxiety Stress Scale

The Depression Anxiety Stress Scale (DASS) is a 21-item questionnaire that measures overall mood. It includes subscales measuring symptoms of depression (Gomez et al., 2014). The questionnaire asks the participant to rate how much a statement applies to them over the past week, for example, “I found it hard to wind down.” Each item is rated on a 4-point scale ranging from “Did not apply to me at all” to “Applied to me very much or most of the “time.” Adding the score from each item multiplying the score by 2 yields a final total score. The DASS-21 is a widely used measure and has excellent reliability and validity for both non-clinical (Coker et al., 2018) and clinical (Antony et al., 1998) populations.

##### Difficulties With Emotion Regulation Scale

The Difficulties with Emotion Regulation Scale (DERS) is a 36-item questionnaire measuring emotion regulation ability (Gratz and Roemer, 2004). The questionnaire asks the participant to rate how often each item applies to them on a 5-point Likert scale ranging from “almost never” to “almost always,” for example, “I am clear about my feelings.” A subset of items are reverse-scored, and subsequently, items are added to produce a total score (range = 36–180) and scores across six subscales: non-acceptance, goal-directed behavior, impulse control, lack of emotional awareness, access to emotion regulation strategies, and lack of emotional clarity. The DERS questionnaire has demonstrated good internal consistency, test-retest reliability, and construct validity (Gratz and Roemer, 2004).

##### Five Facets Mindfulness Questionnaire

The Five Facets Mindfulness Questionnaire (FFMQ) is a 15-item questionnaire assessing trait mindfulness, which is highly related to mental health, and is influenced by exercise participation (Baer et al., 2006). The FFMQ asks the participant to rate how true each statement is of themselves on a 5-point Likert scale ranging from “Never or very rarely true” to “Very often or always true,” for example, “I find myself doing things without paying attention.” Items are added to yield a total score (range = 15–75). The FFMQ has been demonstrated to have sufficient convergent validity, internal consistency, and sensitivity (Gu et al., 2015).

### Procedure

Participants who met eligibility criteria were delivered a Fitbit Inspire HR, Model FB413BKBK (Brooklyn, NY) device *via* a no-contact drop-off protocol to maintain COVID-19 safety requirements mandated by McMaster University. The Fitbit is a smart watch that tracks, amongst other measures, activity level and heart rate. Participants were also scheduled for a virtual pre-assessment with the research team.

#### Virtual Pre-assessment

Virtual pre-assessment sessions were between 9 a.m. and 12 p.m. to minimize time-of-day effects on variability in exercise performance (Chtourou and Souissi, 2012). Participants were requested to refrain from vigorous exercise for 12 h, food for 2 h, and caffeine for 8 h prior to the session. This was verified verbally by asking the participant at the beginning of the virtual pre-assessment. Pre-assessments were held over a secure video teleconferencing link with two members of the research team. Participants provided oral informed consent to participate.

Participants completed a battery of questionnaires to quantify their depressive, anxiety, and other mental health symptoms (see “Questionnaires”). Subsequently, they were provided with a tutorial on how to use and wear their Fitbit device. Participants then were weighed in kilograms using their digital bathroom scale and completed the mCAFT fitness assessment under the supervision of the researchers. Finally, participants were each pseudo-randomly assigned to one of two groups: (1) the exercise group, or (2) the waitlist control group, and provided with instructions for their respective groups.

##### The Modified Canadian Aerobic Fitness Test

The Canadian Society for Exercise Physiology (CSEP)’s Modified Canadian Assessment of Fitness Test (mCAFT) (Weller et al., 1993, 1995) was used as a manipulation check that our exercise intervention was effective at inducing an increase in aerobic fitness. The mCAFT is a submaximal exercise test selected for its accessibility, accuracy, and ease of administration given the virtual environment required by COVID-19-related restrictions including physical distancing. The mCAFT test estimates VO_2_ max, a measure of cardiorespiratory fitness. The test was designed for use at home and is validated for adults aged 15–69 (Weller et al., 1995). Equipment required includes a bathroom scale, a heart rate monitor, closed-toed exercise shoes, and the bottom two stairs of a staircase. Before the test began, 85% of the participants’ predicted maximal heart rate was calculated using the equation 0.85 × (220 - age), to be used as a cut-off to proceed or stop the test. During the test, participants completed one or more 3-min stepping stages using the two bottom steps of their chosen stairs. For each stage, participants listened to the corresponding recording played by the research team, which played music at a clear and specific cadence and instructed participants when to step up or down and with which foot (e.g., “step up left foot,” “step down right foot” etc.). Subsequent stages featured a faster cadence that increased aerobic demand. Once a stage was complete, heart rate was immediately measured using the participant’s Fitbit device. Participants progressed through the stages of the test until their post-exercise heart rate met or exceeded the pre-calculated cut-off. The following predictive equation, widely used in the literature, and originally proposed by Jetté (1979), was used to estimate VO_2_ max: Estimated VO_2_ max (mL⋅kg^–1^⋅min^–1^) = [17.2 + (1.29 × O_2_ cost*)–(0.09 × body weight in kg) – (0.18 × age in years)], where * represents the oxygen cost of stepping during the final completed stage (Canadian Society for Exercise Physiology, 2017).

#### The Exercise Group

Participants assigned to the exercise group completed an 8-week individually delivered aerobic exercise intervention from home, *via* secure video teleconferencing, led by two members of the research team trained in safe and effective delivery of the protocol. The exercise intervention was designed by a CSEP certified personal trainer. The exercise intervention was designed to be fully home-based, free of equipment, and individually delivered, to optimize its accessibility, privacy, and comfort for participants. Exercise adherence is influenced by each of these factors, and prior research indicates that adults affected by mental illness may have specific preferences for activities that can be performed at home, with guidance from an instructor, and with social support (Chapman et al., 2016). Thus, an individualized program design was used to support the potential preferences of the population under study. The exercise intervention consisted of three 40-min exercise sessions per week for 8 weeks. Exercise sessions included a 5-min dynamic warmup, 30 min of exercise, and a 5-min cool down. The 30 min of exercise within each session included five movements performed once each for 30 s, with a 30 s rest interval between each movement. This set of five movements was then repeated for a total of four sets. The movements for each exercise session included a subset of the following: squat variations (squat, sumo squat, squat jump), plank jacks, lunge variations (e.g., side lunge, front lunge), triceps dips, mountain climbers, plank taps, burpees, running in place, snow angels, skaters, jumping jacks, wall sits, push-ups, sitting twists, inchworms, super-mans, glute kicks, triceps push-ups, single leg deadlifts, lying leg raises, dead bugs, and high knees. The research team demonstrated all movements to participants and monitored form through each exercise session. Standard modifications were provided for each movement based on the participant’s perceived intensity. Both less challenging and more challenging modifications were provided as required. Participants wore their Fitbit device for all exercise sessions. Participants reported their heart rate and perceived exertion using the Borg CR-10 scale (Borg, 1982) after each movement within the exercise session to monitor exercise intensity. This measure assesses the perception of how hard the body is working based on physical sensations such as heart rate, breathing rate, and muscle fatigue. The Borg CR-10 is widely used in the field of sports medicine and has demonstrated convergent validity with other scales to assess perceived exertion, such as visual analog scales (Williams, 2017). Participants’ heart rates were monitored such that they remained within a range of 60–75% of predicted maximum heart rate (Tanaka et al., 2001), to stay within a moderate aerobic exercise intensity. More or less challenging modifications were suggested for participants that reported a heart rate outside of this intensity range.

#### The Waitlist Control Group

Participants in the waitlist control group were instructed to remain at their current level of physical activity as reported on the Get Active Questionnaire for the 8-weeks of the study. Participants were administered weekly self-report questionnaires to report their physical activity minutes for that week, as well as qualitatively report any subjective changes in their physical activity level. These participants were subsequently invited to participate in a later cohort of the exercise group; for those controls who later completed the exercise intervention, their data from the exercise component of the study were also included in the exercise group.

#### Virtual Post-assessment

After the 8-week intervention, all participants were scheduled for a virtual post-intervention assessment consisting of the same battery of questionnaires and mCAFT fitness assessment as in the virtual pre-assessment. Participants were then debriefed and compensated as applicable. Upon completion of the exercise group, participants were provided with a digital copy of instructions for all exercise sessions within the intervention, including movement modifications and instructional videos, so that participants could continue to exercise independently if they wish to do so.

### Statistical Analysis

Data were analyzed using IBM SPSS Statistics Software 28. The cutoff for statistical significance was set at *p* < 0.05 (Kim, 2013). Independent samples *t*-tests were used to assess whether there were significant differences between groups at baseline across all measures. Visual inspection using boxplots by group was used to assess equality of variance between groups for *t*-tests (Field, 2013). An independent *t*-test was used as a manipulation check to determine whether our exercise intervention induced the expected changes in aerobic fitness (predicted VO_2_ max). A repeated measures analysis of variance (ANOVA) was used to assess our primary *a priori* hypothesis that exercisers would show greater decreases in mood-related symptoms on the DASS-21 compared to controls. Mauchly’s Test of Sphericity was used to assess whether the assumption of sphericity was met. Secondary outcome measures of trauma symptoms, emotion regulation, and trait mindfulness at pre- vs. post-intervention time points were compared for each group using paired samples *t*-tests corrected for multiple comparisons (critical alpha = 0.016).

## Results

No participants from either the exercise group or the waitlist control group withdrew from the study. All variables were normally distributed based on histogram plots and skewness and kurtosis recommendations. Mauchly’s test of sphericity indicated no issues with sphericity.

### Demographic Characteristics

Demographic characteristics of participants are reported in Table 1. Mean age (standard deviation) in years for the exercise group was 20.7 (0.29). Mean age (standard deviation) in years for the waitlist control group was 21.5 (0.42). An independent samples *t*-test revealed no significant differences in age between the groups [*t*(23) = −1.42, *p* = 0.162].

**TABLE 1:** Demographic characteristics of participants by group.

Baseline characteristic	Exercise group (*N* = 13)	Waitlist control group (*N* = 12)
Gender		
Identify as female	12	11
Identify as male	1	1
Identify as non-binary	0	0
Identify as other	0	0
Mean age (standard deviation) (in years)	20.7 (0.29)	21.5 (0.42)

### Assessing Baseline Differences Between Groups

**FIGURE 1:**
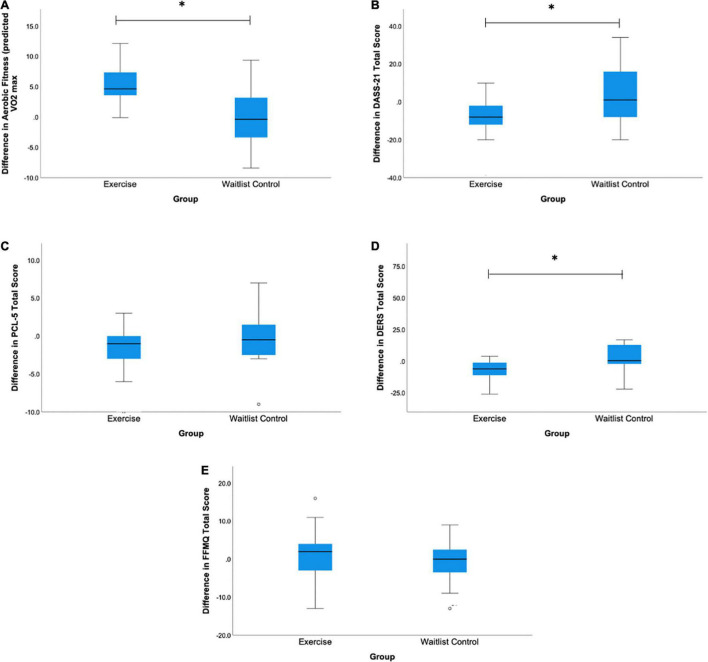
Boxplots of mean difference scores for the exercise and waitlist control groups for **(A)** Aerobic fitness (predicted VO_2_ Max), **(B)** DASS-21 Total Score, **(C)** PCL-5 Total Score, **(D)** DERS Total Score, and **(E)** FFMQ Total Score. Difference scores were calculated by taking the post-intervention score minus the pre-intervention score for each measure for each group. *A significant difference between groups at *p* < 0.05.

Mean pre- and post-intervention scores and standard errors for all measures are reported in Table 2 and displayed as boxplots in Figures 1A–E. Participants in the exercise group and waitlist controls were not significantly different at baseline with respect to aerobic fitness (predicted VO_2_ max) [*t*(23) = −0.721, *p* = 0.478], trauma symptoms [*t*(23) = 0.101, *p* = 0.92], mood symptoms [*t*(23) = −0.258, *p* = 0.79], emotion regulation [*t*(23) = −0.728, *p* = 0.474], or trait mindfulness [*t*(23) = 0.101, *p* = 0.812].

**TABLE 2:** Mean pre- and post- intervention aerobic fitness and mental health-related scores for the exercise and waitlist control groups.

	Exercise group (*N* = 13)		Waitlist control group (*N* = 12)	
Measure	Pre-intervention	Post-intervention	Pre-intervention	Post-intervention
Trauma symptoms (PCL-5 score)	14.00 (1.35)	12.23 (1.47)	13.75 (2.12)	13.25 (1.69)
Predicted VO_2_ max	45.05 (0.97)	49.77 (0.78)	46.20 (1.27)	45.99 (1.08)
Mood symptoms (DASS-21 total score)	31.38 (3.54)	23.07 (3.92)	33.00 (5.27)	37.50 (7.17)
Emotion regulation (DERS total score)	86.58 (5.49)	78.85 (3.95)	80.01 (7.16)	75.73 (5.48)
Trait mindfulness (FFMQ total score)	49.15 (2.10)	48.42 (2.23)	50.08 (2.12)	47.67 (1.79)

*Scores are presented as Mean (Standard error).*

### Manipulation Check: Assessing Differences in Aerobic Fitness Between Groups

Overall, exercisers’ aerobic fitness levels (predicted VO_2_ max) increased from the pre- to post-intervention, while in contrast, control participants’ VO_2_ max changed minimally from pre- to post-intervention (see Table 2 and Figure 1). The exercise intervention was thus effective at inducing the expected improvements in aerobic fitness; those in the exercise group demonstrated a significantly greater increase in aerobic fitness compared to the waitlist control group [*t*(24) = −2.21, *p* = 0.03, Cohen’s *d* = 0.4].

### Primary Research Question: Assessing Differences in Mood Symptoms Between Groups Across Time

A repeated measures ANOVA was conducted to assess the impact of the exercise intervention on mood symptoms. There was a statistically significant difference in mood symptoms from pre- to post-intervention between the exercise and waitlist control groups [*F*(2, 22) = 6.66, *p* = 0.005, partial η^2^ = 0.37]. As can be seen in Table 2, while the exercisers’ mean DASS-21 score dropped by about 8 points, the controls’ DASS-21 score increased by nearly 5 points. Given that our participants’ post-intervention assessment occurred late in their school semester, this is consistent with previous findings that university student participants’ stress and depression levels tend to rise by the end of a school semester, while an exercise intervention is protective against this rise in mood symptoms (Paolucci et al., 2018).

### Secondary Analyses: Assessing Differences in Trauma, Emotion Regulation, and Trait Mindfulness

Pair-samples *t*-tests corrected for multiple comparisons were conducted to assess pre-to-post-intervention changes in our secondary measures of trauma symptoms, difficulties in emotional regulation, and trait mindfulness for each group. While there were no statistically significant differences in trauma symptoms [*t*(12) = 1.80, *p* = 0.09] or trait mindfulness [*t*(12) = −0.41, *p* = 0.688] from pre- to post-intervention for the exercise group, this group did exhibit a significant improvement in emotion regulation [*t*(12) = 3.16, *p* = 0.009] that survived the correction for multiple comparisons (critical *p* = 0.016). In the control group, there were no statistically significant differences in trauma symptoms [*t*(11) = 0.45, *p* = 0.663], emotion regulation [*t*(11) = −0.34, *p* = 0.740], or trait mindfulness [*t*(11) = 0.42, *p* = 0.682], from pre- to post-intervention. As can be seen in Figure 1, while not all changes were statistically significant, the exercise group showed similar overall trends on all measures except mindfulness, with greater improvements in fitness, mood, trauma symptoms and emotional regulation relative to controls.

## Discussion

The current pilot study is the first, to our knowledge, to examine the impact of aerobic exercise on mood symptoms in trauma-exposed young adults presenting with subclinical levels of trauma symptoms. Our 8-week intervention significantly decreased mood symptoms on the DASS-21, and improved emotion regulation difficulties on the DERS scale, in the exercise group compared to the waitlist control group. Our results align with previous research among individuals with PTSD, where physical activity reduced depressive symptoms (Rosenbaum et al., 2015b). This suggests that exercise may be an effective adjunctive or alternate treatment option to improve mood and thus help to manage and prevent further decline in mental health overall in those with subclinical trauma symptoms. This research serves as a pilot for future planned research investigating the impact of exercise interventions for those experiencing symptoms related to trauma exposure and those at-risk for developing PTSD.

While aerobic exercise has a large body of research supporting its efficacy for improving mood (Moses et al., 1989; Byrne and Byrne, 1993; Babyak et al., 2000; Mota-Pereira et al., 2011; Paolucci et al., 2018), our results extend these findings to young adults who have had trauma exposure but have not been diagnosed with PTSD, and thus who may be at risk for the development of PTSD or additional mental health concerns. This provides rationale for encouraging the inclusion of exercise in clinical recommendations for trauma-exposed populations with subclinical PTSD symptoms. Critically, having subclinical trauma symptoms puts individuals at increased risk for developing PTSD (Mylle and Maes, 2004). Moreover, those experiencing subclinical trauma symptoms often display similar levels of distress and impairments in social and occupational functioning compared to those with a PTSD diagnosis (Alim et al., 2006; Korte et al., 2016; Artime et al., 2019). Thus, our findings provide further support for the notion that exercise assists in managing depressive symptoms in trauma-exposed populations. Moreover, combing our findings with previous literature, exercise may have the potential to help prevent the development of a comorbid mood disorder and/or a transition to clinical levels of PTSD.

We also observed improvements in emotion regulation in the exercise group only. This aligns with prior research indicated that engaging in exercise benefits emotion regulation skills Bernstein and McNally, 2018. This makes sense with respect to the observed improvements in mood in the exercise group, given that emotion regulation skills are associated with more positive mood (Hu et al., 2014). While we did not observe statistically significant improvements in trait mindfulness or reductions in trauma symptoms, a trend can be observed in the data indicating improvement in trauma scores in the exercise group relative to the waitlist control group (see Figure 1C). A replication of our study is warranted to see whether this trend might be significant with a larger sample size. Alternatively, it is possible that our chosen exercise prescription was not sufficient in time, duration, or mode to induce changes in these measures. Indeed, interventions of 8 weeks in length are on the shorter side of interventions identified as being effective at reducing anxiety in healthy population and in those with anxiety disorders, with most interventions lasting 10–12 weeks (Herring et al., 2010). Similarly, studies indicating changes in trait mindfulness have used 12 weeks long exercise programs (Mothes et al., 2014). However, it is important to note that our study sample of young adult students is unique in that students are typically exposed to greater amounts of anxiety and stress as their academic semester progresses (Pitt et al., 2018), which coincided with the progression of our 8-week exercise intervention. Thus, given that the combined stress, depression, and anxiety (DASS) scores improved in the exercisers but worsened in the controls, it appears that our exercise intervention may have buffered against expected increases in stress that typically occur within an academic semester.

The current study used a novel and innovative exercise intervention format that catered to the unique needs of our participants during the COVID-19 pandemic. In general, individuals with trauma symptoms exhibit lower levels of participation in sports and physical activities (de Assis et al., 2008). Moreover, individuals with mental illness often have specific preferences in terms of exercise participation. For example, adults diagnosed with a mental illness appear to prefer physical activities that can be performed at or close to home, are guided, and completed with social support from others of similar physical ability (Chapman et al., 2016). Thus, individuals with ongoing mental health issues may be reluctant to go to a gym-based environment on their own or join a group exercise class. Furthermore, such facilities have been shut down in many regions throughout most of the pandemic. However, with supportive, home-based individual instruction, those with trauma exposure at risk of developing a mood disorder may be encouraged by the mood-related benefits of physical activity and increase their exercise participation in the long-term. Importantly, the current study employed a home-based program designed for beginners and delivered online with supportive, highly trained researchers that were able to tailor each exercise session to the participant’s needs. Barriers to participation were decreased by designing a program requiring no equipment and in a one-on-one setting such that concerns about self-perception or judgment from others were minimized. This likely contributed to the zero drop-out rate; no participants from either group withdrew from the study, indicating that the exercise intervention was not only appropriate and tolerable but may have suited the needs of this population and allowed them to maintain their exercise participation.

The current pilot study had a number of limitations, most importantly including the relatively small sample size. For that reason, we also included data from wait-listed controls who subsequently participated in the exercise intervention in the exercise group. A larger sample size and replication of our initial results are required to lend further support for these findings. In addition, our sample consisted of young adult university students, and therefore more research is needed to elucidate whether our findings apply equally to at-risk youth and adolescents or those at middle to advanced ages. Future research should also collect additional measures associated with trauma history, such as socioeconomic status and adverse childhood experiences, to provide a fuller analysis of factors that may impact the effect of exercise on mental health in this population. Further, exercise as an intervention strategy for both trauma-exposed populations as well as those with PTSD should be directly compared to other standard and alterative treatment options, such as mindfulness-based intervention programs, cognitive processing therapy, and prolonged exposure, to examine the unique characteristics of exercise and its impact on mood and psychological health. Similarly, research that directly compares the impact of exercise on those with a clinical diagnosis of PTSD and those with trauma exposure and subclinical symptoms is required to understand how exercise may differentially impact symptoms and meet unique needs of each group.

While research regarding exercise and PTSD is in its early stages, our findings align well with the current state of the literature, which points to an important role for exercise in addressing symptoms of depression, PTSD, and accompanying physical health concerns. Long-term, longitudinal studies are required to formally examine how physical activity participation may be protective against the development of PTSD in those with subclinical symptoms. In addition, determining the specific components of exercise, such as type, dose, and setting, that are most suitable for managing depressive symptoms in subclinical and clinical PTSD is vital for designing and integrating exercise into primary and secondary treatment recommendations for these populations.

## Data Availability Statement

The datasets presented in this article are not readily available as per our REB-approved protocol. Requests to access the datasets should be directed to the corresponding author.

## Ethics Statement

The studies involving human participants were reviewed and approved by the McMaster Research Ethics Board. The patients/participants provided their written informed consent to participate in this study.

## Author Contributions

AM, MM, and SB designed the research questions and methods and edited the manuscript for the final submission. AM planned the study, collected the data, analyzed the data, and wrote the manuscript. AM and SB interpreted the results. All authors contributed to the article and approved the submitted version.

## Conflict of Interest

The authors declare that the research was conducted in the absence of any commercial or financial relationships that could be construed as a potential conflict of interest.

## Publisher’s Note

All claims expressed in this article are solely those of the authors and do not necessarily represent those of their affiliated organizations, or those of the publisher, the editors and the reviewers. Any product that may be evaluated in this article, or claim that may be made by its manufacturer, is not guaranteed or endorsed by the publisher.
